# Detection of Human cytomegalovirus *UL55* Gene and IE/E Protein Expression in Colorectal Cancer Patients in Egypt

**DOI:** 10.1186/s12885-023-11200-x

**Published:** 2023-08-03

**Authors:** May Raouf, Ahmed A. Sabry, Mahinour A. Ragab, Samar El Achy, Amira Amer

**Affiliations:** 1 Medical Microbiology and Immunology Department, Faculty of Medicine, Alexandria University, 0 Khartoum Square, Azarita Medical Campus, Alexandria, 21131 Egypt; 2 General Surgery Department, Faculty of Medicine, Alexandria University, Alexandria, Egypt; 3 Pathology Department, Faculty of Medicine, Alexandria University, Alexandria, Egypt

**Keywords:** Human cytomegalovirus, Colorectal cancer, *UL55* gene, HCMV IE/E proteins, Immunohistochemistry

## Abstract

**Background:**

A possible relation between Human cytomegalovirus (HCMV) and colorectal cancer (CRC) has been widely explored with an unclear role yet speculated.

**Aim:**

The study aimed at detecting HCMV *UL55* gene, immediate early and early (IE/E) proteins in colorectal tumor tissues and adjacent non neoplastic tissues (ANNT). Also, it aimed to correlate HCMV presence with CRC clinicopathological features.

**Subjects and methods:**

A prospective study of 50 HCMV seropositive patients with resectable CRC were enrolled in the study. Demographic, clinical, and radiological findings were recorded. Pathological assessment was done. Paired CRC tumorous and ANNT were examined for HCMV *UL55* by PCR and for IE/ E proteins by immunohistochemistry (IHC).

**Results:**

70% of CRC patients enrolled were females and 36% were elderly (> 60y). Adenocarcinoma was the prevalent histopathological type (92%) with Grade 2, higher stages, and nodal involvement accounting for (64%, 64% and 56%) respectively.

HCMV detection was significantly higher in tumoral tissue versus ANNT by PCR and IHC (*P* < 0.001, *P* < 0.008) respectively. Moderate agreement was found between the two techniques (κ = 0.572, *P* < 0.001).

Univariate analysis identified HCMV presence to be significantly higher in elderly patients, in tumors with higher stage and with nodal involvement (*P* = 0.041,* P* = 0.008,* P* = 0.018 respectively). In multivariate analysis, the latter two retained significance (*P* = 0.010,* P* = 0.008).

**Conclusion:**

CRC tumor tissues are more infected by HCMV than ANNT. A significant association of HCMV presence with a higher CRC tumor stage and nodal involvement in an age-dependent manner was detected. HCMV oncomodulatory and a disease progression role is suspected.

**Supplementary Information:**

The online version contains supplementary material available at 10.1186/s12885-023-11200-x.

## Introduction

Human cytomegalovirus (HCMV) is a ubiquitous virus with population seropositivity reaching up to 100% in Africa and Asia [1]. It attains a lifelong latency in haematopeitic progenitor cells. HCMV causes a wide range of infections ranging from asymptomatic to mild infections and occasionally severe infections in the immunocompromised as well as teratogenicity during pregnancy [2]. Correlation between HCMV and different human cancers has been widely explored with mixed results between studies [3].

HCMV encodes a wide range of proteins which have major oncomodulatory effect on changing cell survival behavior and alteration of immune surveillance [4]. HCMV *US28* gene is implicated in the HCMV-generated angiogenic phenotype by secreting vascular endothelial growth factor (VEGF), a key molecular for angiogenesis [5, 6]. HCMV encodes Immediate early (IE) proteins including UL37 exon 1, UL36, and UL38 proteins that interfere with antiapoptotic genes e.g. retinoblastoma, p53 and cyclins as well as induction proapoptotic genes e.g. *fos* and *myc* [7]. Also, the level of Cyclooxygenase 2 (COX-2) increases in viral infected cells. COX-2 is a main source of prostaglandins which promotes inflammatory conditions [8]. Moreover, HCMV infection changes the formulation of matrix metalloproteinases, that enhance tumor growth by destroying matrix barriers and increasing angiogenesis, thus are crucial in cancer metastasis*.* Chromosomal aberration and instability are also described [9, 10]. Moreover, HCMV impacts the anticancer immunity. It enables tumors to evade immune surveillance by encoding viral proteins as US28, UL111A, and UL144 resulting in HCMV-induced immunological tolerance, which promotes tumor growth [11, 12]. Additionally, HCMV has been recently categorized into low risk (LR) strains causing oncomodulation and high risk (HR) strains with direct oncogenic potential. The molecular mechanisms behind HCMV-induced oncogenesis are complex, with cellular stress, PGCCs (Polypoid giant cancer cells), and genomic instability all playing a role [11].

HCMV can infect any part of the gastrointestinal tract from the mouth till the rectum with a spectrum of illnesses. Colorectal cancer (CRC), a multifactorial disease with infectious agents incriminated, is a leading cause of cancer with high morbidity and mortality worldwide [13].

Molecular based methods are the most widely used diagnostic tests for detection of HCMV infection as well as detecting reactivation [14]. Immunohistochemistry (IHC) for demonstration of HCMV proteins in tissue specimens is not routinely done but can give important topographical information about the viral location inside the cell [15].

The study aimed at detecting HCMV *UL55* gene, immediate early and early (IE, E) proteins in colorectal tumor tissues and the adjacent non neoplastic tissues (ANNT). Also, it aimed to correlate HCMV presence with clinical and pathological features of the disease. To our best knowledge, this is the first study exploring HCMV and CRC relationship in Egypt.

## Patients and methods

### Study setting and exclusion criteria

A prospective study of 50 HCMV seropositive patients with resectable CRC admitted to the General Surgery department for tumor resection from December 2020 to May 2022 was carried out.

Patients aged younger than 20 years or pregnant, had otherwise immunocompromising conditions, patients who received previous neoadjuvant chemotherapy, or with metastasis were excluded.

### Data collection and Clinical assessment

Demographic data, medical, drug, surgical, family history as well as patients’ complaints and clinical signs were recorded.

### Preoperative investigations were done including

A. Laboratory tests (Complete blood picture, and carcinoembryonic antigen (CEA)) [16]
B. Radiological investigations (MSCT Enterocolonography scan to access resectability)
C. Colonoscopy and biopsy pathology report [17]

### Surgically resected CRC specimens were subjected to: Routine pathological processing [17]

The specimens were fixed in 10% buffered formalin and grossed the following day, with representative sections from the tumor, surgical margins, and pericolic lymph nodes being taken for proper histopathological assessment, grading and staging.

### Conventional PCR for detection of HCMV DNA [18, 19]

Fifty paired CRC tumor specimens and their ANNT (> 5 cm apart) were examined for the presence of HCMV DNA by PCR. Excessive formalinization and paraffin embedding were avoided to preserve the integrity of genetic material. Paired tissues were stored at -80ºc.

### DNA extraction

DNA was extracted from 25 mg of tissue using QIAamp® DNA FFPE tissue according to manufacturer 's instructions (DNA Mini Kit, Qiagen, CA, USA). The quantity of DNA was confirmed by NanoDrop spectrophotometer. Two primers for amplification of HCMV *UL55*, encoding a highly conserved gB transmembrane protein, were selected:

5' GCGGTGGTTGCCCAACAGGA 3' and 3' ACGACCCGTGGTCATCTTTA 5' [14]

Conventional PCR reaction was done using Thermoscientific DreamTaq Green PCR Master Mix according to manufacturer's instructions. Briefly, A 25 μl total reaction volume comprising 12.5 μl DreemTaq Green PCR Master Mix, 1 μl downstream primer (10 pmol), 1 μl upstream primer (10 pmol), 1 μl DNA and 9.5 μl H2O was used. The following amplification conditions were followed in an orderly manner based on primer blasting: Activation at 95 °C for 3 min then a total of 40 cycles were done, denaturation at 95 °C for 30 s, annealing at 55 °C for 30 s, then extension at 72 °C for 60 s. A final extension step at 72 °C for 10 min was done.

#### Gel electrophoresis

The amplification products were analyzed by 2% agarose gels followed by visualization using an ultraviolet trans-illuminator. The presence of a band of 94 base pairs (bp), compared to a reference sizing ladder of known fragment length, was considered positive for HCMV DNA [14].

### Immunohistochemistry [15]

Immunohistochemical staining on formalin-fixed, paraffin-embedded (FFPE) sections was carried out according to manufacturer’s instructions using the following monoclonal antibodies: anti-CMV (clones CCH2 and DDG9, dilution 1:200, (DAKO, Carpinteria, CA, USA), which contains two antibodies that react specifically with a 76 kDa HCMV early protein and an immediate early DNA binding protein p52.

#### Specimen preparation

The antibody was used for labeling FFPE tissue sections. Tissue specimens were cut into sections of approximately 4 µm. Pre-treatment with heat-induced epitope retrieval (HIER) was required using Dako PT Link (Code PT100/PT101). Optimal results were obtained by pretreating tissues using EnVision FLEX Target Retrieval Solution, High pH (50x) (Code K8000/K8004).

Immunoreactions were developed using the EnVision FLEX, High pH, (Link) (Code K8000), using a biotinylated secondary antibody. DAB (Thermo Scientific CAT# 34,065) was used as a chromogenic dye and the tissue was counterstained with Mayer’s Hematoxylin.

Specimens were considered positive if they showed positive staining within the epithelial cells, and negative if they failed to show positive staining within the epithelial cells. Correlation with the viral cytopathic effects was an indispensable confirmatory tool utilized in most cases.

### Statistical analysis [20]

Data was fed to the computer and analyzed using IBM SPSS software package version 20.0***.* (**Armonk, NY: IBM Corp**)**^.^ Numerical data were represented as *n* (%). Chi-square test with Fisher’s Exact or Monte Carlo correction were used to compare different groups with categorical variables. Cohen's Kappa coefficient was used to assess the agreement between PCR and IHC. Univariate values of *P* < 0.05 were followed by multivariate analysis. The significance of the obtained results was judged at the 5% level.

## Results

### Patients’ characteristics, clinical and laboratory findings

Among the 50 resectable CRC studied patients, 35 (70%) were females and 18 patients (36%) were elderly (> 60 y). Most patients presented with abdominal pain followed by weight loss and diarrhea (74%,72% and 70% respectively). Intestinal obstruction was present in 10 patients (20%). Most patients had more than one symptom. Family history of colorectal cancer, or previous intestinal history of polyps were recorded in 6 patients (12%). Anaemia was detected in 36 patients (72%) and serum CEA was elevated in 12 (24%).

### Pathological assessment of resected colorectal cancer

More than half of the cases (52%) had sigmoid cancer. Adenocarcinoma was the predominant histopathological type (92%) and 62% of tumors were larger than 5 cm in maximum dimension. Moderately differentiated, grade 2 tumors, higher stages (pT3 + pT4) and nodal involvement accounted for 64%, 64% and 56% respectively among different tumoral grades and stages. None of the tumors exhibited distant metastasis.

### HCMV DNA detection by PCR

**Fig. 1:**
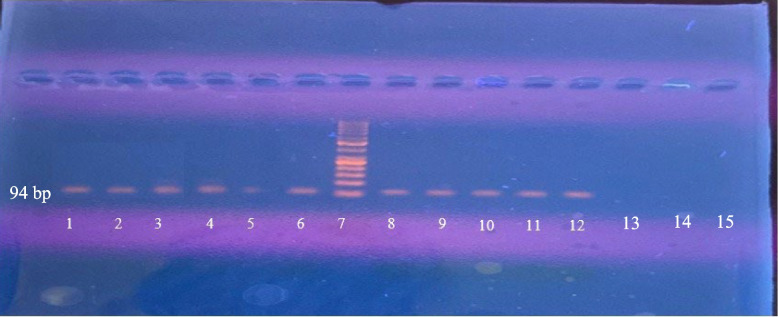
PCR analysis for HCMV UL55 gene detection (94-bp) on 2% agarose gel. Lane 7: 50 bp DNA ladder, Lane 8: positive control, Lanes 1–6: TTs showing positive PCR results, Lanes 9–12: ANNTs showing positive PCR results, Lanes 13–15: TTs showing negative results. PCR: Polymerase chain reaction, HCMV: Human cytomegalovirus, bp: base pairs TTs: Tumor tissues, ANNTs: Adjacent non neoplastic tissues

Among the 50 tumorous specimens, HCMV *UL55* gene was detected in 32 (64%) samples, while only 13 (26%) samples of ANNT showed a positive result. A statistically significant difference was detected between the two groups (P < 0.001) (Table 1, Fig. 1). Tissues extracted DNA purity, concentration and PCR results are shown in Table S1.

**Table 1:** Comparison between Tumorous and Adjacent non- neoplastic tissue regarding the detection of Human cytomegalovirus DNA by Polymerase Chain Reaction

**HCMV DNA**	**Tumor Tissue (*n* = 50)**		**ANNT (*n* = 50)**		**χ**^2^	***P***
	**NO**	**%**	**No**	**%**		
Positive	32	64.0	13	26.0	**14.586**	< 0.001^*^
Negative	18	36.0	37	74.0		

*ANNT* Adjacent non- neoplastic tissue, χ^2^ Chi Square test

*p* value for comparing between tumor and ANNT

^*^Statistically significant at 5% level of significance

### Immunohistochemical staining

**Fig. 2:**
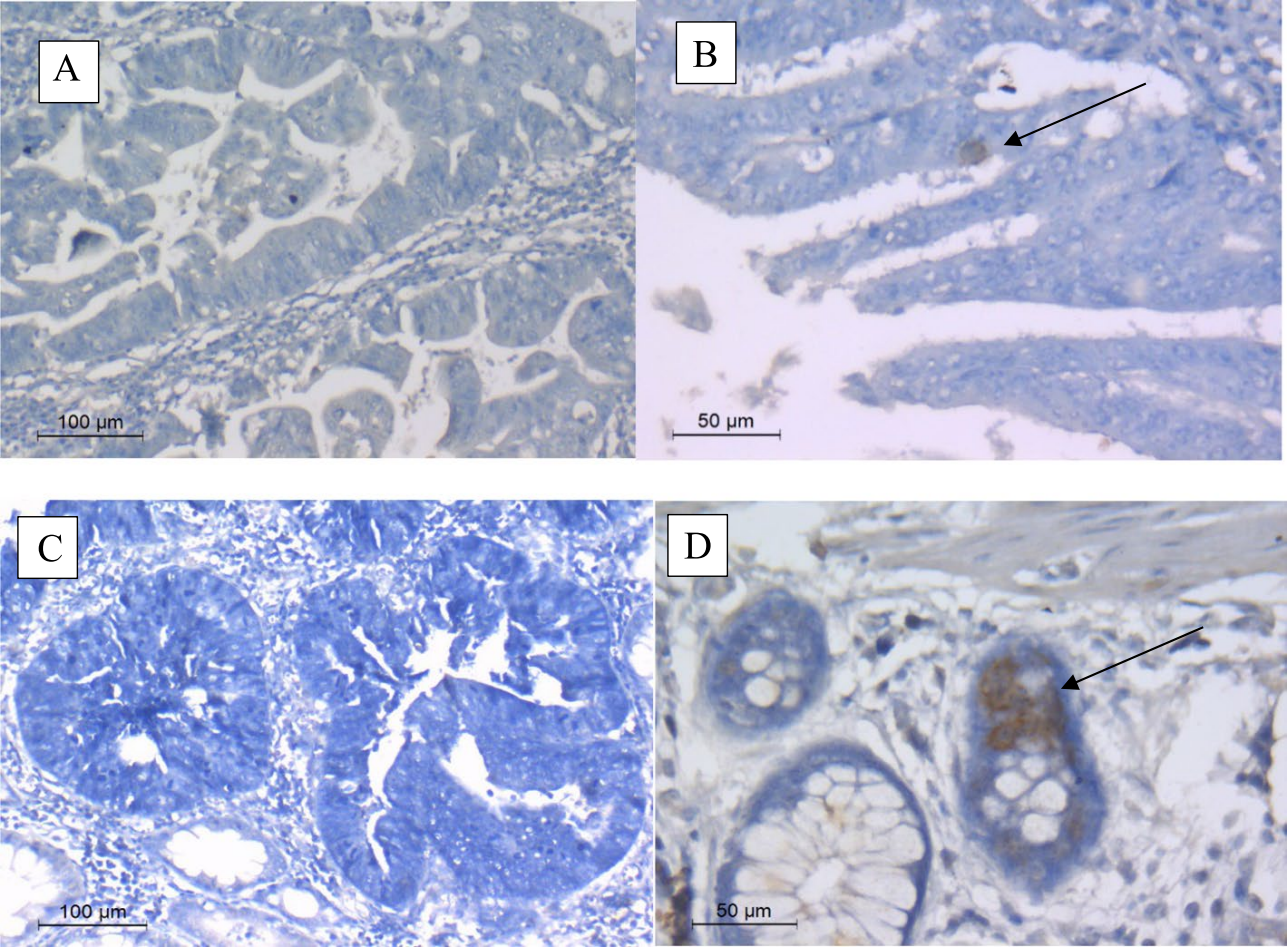
Photomicrography of **A** IHC staining of adenocarcinoma with negative result, **B** IHC staining of adenocarcinoma with positive result, **C** IHC staining of adenocarcinoma with no assessment due to increased N/C ratio, **D** IHC staining of ANNT with positive result

IHC was carried out on 50 paired FFPE sections**.** Among the 50 tumorous specimens, IHC was positive in 14 samples (28%), negative in 15 (30%) while 21 (42%) samples could not be assessed due to increased N/C ratio. Ten (20%) samples of ANNT demonstrated a positive result while 40 (80%) were negative. IHC results was significantly different between the two groups (*P* < 0.008) (Table 2 and Fig. 2A-D).

**Table 2:** Comparison between Tumorous and Adjacent non- neoplastic tissue (*n* = 50) regarding detection of Human cytomegalovirus Immediate Early and Early proteins by Immunohistochemistry

**IHC**	**Tumor tissue (*n* = 29)**		**ANNT (*n* = 50)**		**χ**^2^	***p***
	**No**	**%**	**No**	**%**		
**Positive**	**14**	**28.0**	**10**	**20.0**	33.030*	0.008^*^
**Negative**	**15**	**30.0**	**40**	**80.0**		
**Cannot be assessed**	**21**	**42.0**				

*IHC* Immunohistochemistry, *ANNT* Adjacent non- neoplastic tissue, χ^2^ Chi Square test

*p* value for comparing between tumor and ANNT

^*^Statistically significant at 5% level of significance

### Concordance between Polymerase Chain Reaction and Immunohistochemistry results

Correlation between PCR results and IHC was evaluated in clearly assessed tumorous (*n* = 29) and ANNT (*n* = 50). Matched positive results by PCR and IHC was detected in 21 specimens (14 tumor tissue and 7 ANNT) and matched negative results was detected in 42 specimens (8 tumor tissue and 34 ANNT). IHC showed negative results in 13 PCR positive specimens (7 tumor tissue and 6 ANNT). Conversely, only three ANNT specimens were positive by IHC whilst being negative by PCR. Moderate agreement between PCR and IHC in detecting HCMV was detected (Kappa coefficient = 0.572, *P* < 0.001) (Table 3).

**Table 3:** Human cytomegalovirus detection in Tumorous and Adjacent non-neoplastic specimens from colorectal cancer patients

**IHC**	**PCR**		Cohen Kappa Coefficient
	HCMV Positive	HCMV Negative	
HCMV Positive	21	3	Kappa = 0.572 *P* < 0.001*
HCMV Negative	13	42	

*PCR* Polymerase Chain Reaction, *IHC* Immunohistochemistry, *HCMV* Human Cytomegalovirus

^*^Statistically significant at 5% level of significance

### Correlation between demographic and clinicopathological data versus HCMV DNA results

Different demographic and clinicopathological parameters of the studied patients were correlated to HCMV DNA detection. A significantly statistical difference was detected in patients aged > 60 years, higher CRC stages (T3 and T4) and tumors with nodal involvement (*P* = 0.033, *P* = 0.006, *P* = 0.015) respectively (Table 4).

**Table 4:** Demographic characteristics and clinicopathological parameters of studied CRC patients in relation to detection of Human cytomegalovirus DNA by PCR

**Characteristic**	**Overall 50 (100%)**		**HCMV DNA detected 32 (64%)**		**HCMV DNA Non detected 18 (36%)**		χ^2^	***p*-value**
	**No**	**%**	**No**	**%**	**No**	**%**		
**Age (years)**								
≤ 60	32	64.0	17	53.1	15	83.3	4.563^*^	0.033^*^
> 60	18	36.0	15	46.9	3	16.7		
**Gender**								
Male	15	30.0	12	37.5	3	16.7	2.381	0.123
Female	35	70.0	20	62.5	15	83.3		
**Chronic illness**								
Diabetes	16	32.0	12	37.5	4	22.2	1.236	0.266
Hypertension	15	30.0	11	34.4	4	22.2	0.810	0.368
Other chronic diseases	10	20.0	9	28.1	1	5.6	3.668	^FE^*p* = 0.073
**Family history of colorectal cancer**	3	6.0	2	6.3	1	5.6	0.010	^FE^*p* = 1.000
**History of polyps**	3	6.0	2	6.3	1	5.6	0.010	^FE^*p* = 1.000
**T**								
Lower Stage (I + II)	18	36.0	7	21.9	11	61.1	7.697^*^	0.006^*^
Higher Stage (III + IV)	32	64.0	25	78.1	7	38.9		
**N**								
No nodal involvement	22	44.0	10	31.3	12	66.7	5.864^*^	0.015^*^
Nodal involvement	28	56.0	22	68.8	6	33.3		
**Tumour size**								
< 5 cm	19	38.0	13	40.6	6	33.3	0.260	0.610
> 5 cm	31	62.0	19	59.4	12	66.7		
**Pathology histologic grade**								
Non gradable	1	2.0	1	3.1	0	0.0	3.268	^MC^*p* = 0.333
G1	16	32.0	12	37.5	4	22.2		
G2	32	64.0	19	59.4	13	72.2		
G3	1	2.0	0	0.0	1	5.6		
**Pathology tumor site**								
Cecum	11	22.0	6	18.8	5	27.8	8.466	^MC^*p* = 0.220
Rectum	4	8.0	4	12.5	0	0.0		
Sigmoid	26	52.0	17	6	9	50.0		
Splenic flexure	1	2.0	0	0.0	1	5.6		
Ascending colon	2	4.0	2	6.3	0	0.0		
Descending colon	2	4.0	2	6.3	0	0.0		
Hepatic flexure	3	6.0	1	3.1	2	11.1		
Whole colon	1	2.0	0	0.0	1	5.6		

χ^2^ Chi Square test, *MC* Monte Carlo, *FE* Fisher Exact

*p* value for comparing between HCMV detected and HCMV non detected

^*^Statistically significant at 5% level of significance

### Univariate and Multivariate binary logistic regression for parameters correlated with HCMV DNA presence in tumoral tissue of the studied patients

Univariate analysis detected significant HCMV DNA presence in elderly patients, CRC with higher stages and nodal involvement.

(OR:4.412, 95% CI:1.066 – 18.266, *P* = 0.041), (OR:5.612, 95% CI:1.584 –19.886, *P* = 0.008), (OR:4.400, 95% CI:1.283 – 15.091, *P* = 0.018) respectively.

in the multivariate analysis, significant HCMV DNA detection was retained in CRC with higher stages and nodal involvement (OR:9.462, 95% CI:1.709 – 52.375, *P* = 0.010) (OR:10.046, 95% CI: 1.808 – 55.838, *P* = 0.008) respectively Table 5.

**Table 5:** Univariate and Multivariate binary logistic regression for parameters correlated with HCMV DNA presence in tumoral tissue of the studied patients

	**Univariate**		^**#**^**Multivariate**	
	***p***	**OR (LL – UL 95%C.I)**	***p***	**OR (LL – UL 95%C.I)**
**Age (> 60)**	0.041^*^	4.412^*^(1.066 – 18.266)	**0.083**	4.287 (0.828 – 22.190)
**Higher T Stage (III + IV)**	0.008^*^	5.612^*^(1.584—19.886)	0.010^*^	9.462^*^(1.709—52.375)
**Nodal involvement**	0.018^*^	4.400^*^(1.283 – 15.091)	0.008^*^	10.046^*^(1.808—55.838)

*OR* Odd`s ratio, *C.I* Confidence interval, *LL* Lower limit, *UL* Upper Limit

^#^All variables with *p* < 0.05 were included in the Multivariate analysis

^*^Statistically significant at 5% level of significance

## Discussion

The relation between HCMV and CRC pathogenesis is of a high research interest and being increasingly explored aiming to unravel the precise viral role. The aim of the current study was to detect HCMV by PCR and IHC in colorectal cancer samples as well as to correlate HCMV presence to different CRC demographic, clinical and pathological parameters.

Overall, Colorectal cancer is the second leading cause of cancer mortality and third in incidence. The incidence of CRC is rising in low-medium income countries (LMIC) due to changes in lifestyle [21]. HCMV is highly ubiquitous reaching almost 100% seroprevalence in adults in developing countries with a lifelong latency [1, 22]. In the current study, HCMV presence was assessed in paired tumorous and ANNT tissues of 50 seropositive CRC patients by two techniques; PCR and IHC. *UL55* gene primer, encoding highly conserved HCMV gB, was chosen according to a HCMV primer comparative study which reported PCR sensitivity to be inversely proportional to the length of the PCR product i.e. sensitivity being higher when the product is less than 100 bp [14]. DNA was extracted from tissues with minimal formalinization and before fixation to preserve DNA integrity [19]. A significantly higher detection rate was observed in PCR results in tumorous tissues versus ANNT group (64% vs 26%, *P* < 0.001). Two metaanalyses, one investigating HCMV presence in gastrointestinal cancers and the other focusing on CRC, significantly detected HCMV in tumoral tissues [23, 24]. On the other hand, recently Chelbi et al*.* showed no difference of *UL55* gene detection in tumor versus peritumor tissues [25]. Also, earlier studies found no relation between HCMV and CRC [26–28]. This might be due to the small number of included cancerous samples, non-optimization of PCR technique, or due to testing FFPE as tumor specimens which was avoided in this study. HCMV infection at certain stages of tumor development followed by its clearance is also a possible cause.

IHC is a valuable technique to detect viral protein expression and thus differentiate latent from active infection as well as demonstrate the specific site inside the cell. HCMV IE/E proteins are transactivators for expression of early and late viral genes. They are essential to establish lytic infection and viral re-activation dysregulating cellular functions [5, 29].

In the present study, a significantly higher detection of HCMV IE and E proteins was observed in tumorous tissues versus ANNT group (*P* < 0.008). Taher et al*.* reported in 2 studies higher prevalence of HCMV IE DNA and protein expression in CRC and breast cancer and their metastases to the brain with poor outcome [30, 31]. Recently, two studies reported that HCMV gB DNA detection or expression of HCMV IE in breast cancer was found to be associated with shorter survival [32, 33]. HCMV IE and E proteins were always detected in the cytoplasm in this study. Chen et al*.* and Dimberg et al*.* also demonstrated that viral antigens are preferentially detected in the tumor cytoplasm [34, 35]. Two possible explanations were hypothesized for HCMV viral protein expression in tumoral tissue, either HCMV is carried to the tumor and become reactivated due to suppressed immunity or, more likely, the inflammatory process reactivates the already existing virus [18].

In the present work, a lower HCMV detection rate was obtained by IHC than PCR which could be due to expression of a mutated protein, [36] or due to masking viral proteins by increased N/C ratio in malignant cells which at many times reached a ratio of 1:1.

When comparing PCR and IHC results, a moderate agreement was detected (Kappa coefficient = 0.572, *P* < 0.001). One study comparing PCR and IHC to explore the relation between HCMV and breast cancer showed a fair concordance (kappa = 0.345; *P* = 0.003) [37]. In fact, combination of PCR and IHC to explore HCMV role in CRC in Egyptian population and reporting the significant association between HCMV presence and CRC as well as detection of the moderate agreement between the two techniques is distinctive in the current study. Only a few earlier studies combined IHC to PCR for HCMV detection and found no relation to CRC. Interestingly, the fact that immunostaining was detected in the cytoplasm and not in the nucleus, as in cases of acute infection, was their reason to consider it as nonspecific staining [26, 28]. Nevertheless, “the hit and run” theory of oncogenesis could not be ruled out. On the contrary, a pioneer study by Harkins et al*.* detected a significant relationship and postulated an oncogenic rule [38]. A recent study of the effect of IE viral protein on CRC derived stem cell discovered cytoplasmic localization to be the most commonly detected [39].

In the present study, 70% of the enrolled CRC patients were females and 36% were elderly (> 60y). A recent meta-analysis reported higher incidence of CRC in males, and in elderly patients [40]. Adenocarcinoma was the prevalent histopathological type (92%) with Grade 2, higher stages (T3 + T4) and nodal involvement accounting for (64%, 64% and 56%) respectively. Our findings could be attributed to scarcity of screening programs so that the disease is only discovered after seeking medical advice on complaining.

When demographic and clinicopathological parameters were correlated to HCMV DNA presence, a statistically significant relationship in patients aged > 60 years, with higher CRC stages and tumors with nodal involvement (P = 0.033, P = 0.006, P = 0.015) respectively.

In multivariate analysis, significant HCMV DNA detection was retained in CRC higher stages (T3 + T4) and nodal involvement (*P* = 0.010,* P* = 0.008). Thus, the most independent factor affecting HCMV detection was nodal involvement followed by stages 3 and 4 in colorectal cancer. This study’s results thus suspect a HCMV role in disease progression even in the absence of a clear role.

In a study of CRC progression in the elderly, Chen et al*.* demonstrated poorer outcome in the presence of HCMV independent of other factors and reported no correlation with demographic, clinical or pathological factors as grading, local extension and nodal involvement to HCMV presence. They suggested that decreased immunity with age may be an important risk factor to HCMV reactivation, nevertheless, a causal relation could not be confirmed [36]. In another study for the non-elderly, Chen et al*.* found an opposite effect suggesting a dual oncomodulatory age-dependent effect for HCMV presence [41]. This may be due to stimulation of the immune response to HCMV infection in young age [42].

Interplay between oncomodulatory/oncogenesis role of HCMV was suggested in the context of identification of high risk HCMV strains versus low risk strains showing difference in disease progression, survival rate and response to treatment [5, 43]. Surprisingly, on the other hand, a recent clinical analysis study detected a statistically significant correlation between HCMV infection and lower CRC incidence [44]. The detection of HCMV DNA as well as active transcription of IE protein in CRC tumor tissues points to a substantial oncomodulatory profile in the elderly.

## Conclusion

CRC tumor tissues are more infected by HCMV than ANNT. A significant association of HCMV presence with a higher CRC tumor stage and nodal involvement in an age-dependent manner was detected. HCMV oncomodulatory and a disease progression role is suspected.

### Limitations and recommendations of the study

A larger follow up study with stratification according to demographic and clinicopathological parameters is needed. Alleged viral proteins responsible for oncogenesis should be studied. Treatment regimens including HCMV antivirals will be very valuable to assess the intensity of HCMV role. The study findings combined with other multicenter studies would help expert panel to develop guidelines regarding HCMV prophylaxis and/or add on antiviral therapy that can improve survival rate.

### Supplementary Information

**Additional file 1:** **Table S1.** Tissues extracted DNA purity, concentration and PCR results.

## Data Availability

Available upon reasonable request from the corresponding author.
